# Vitrification of bovine oocytes and embryos: roadmaps to cryopreservation success

**DOI:** 10.1590/1984-3143-AR2026-0060

**Published:** 2026-08-31

**Authors:** Teresa Mogas

**Affiliations:** 1 Departament de Medicina i Cirurgia Animals, Universitat Autònoma de Barcelona, Cerdanyola del Vallès, Barcelona, Spain

**Keywords:** cryotolerance, mathematical modelling, extracellular vesicles, direct transfer, oxidative stress

## Abstract

Cryopreservation of bovine oocytes and *in vitro* produced (IVP) embryos represents a central tool for the global distribution of genetic material and the efficient application of assisted reproductive technologies. Although vitrification has become the preferred alternative to slow freezing because it avoids ice crystal formation, consistent and reproducible results are still difficult to achieve, especially in oocytes. Most vitrification protocols have traditionally been developed through empirical “trial and error” approaches, with limited consideration of the biological and biophysical factors that determine cryosurvival. Current progress, however, points toward a more rational framework based on membrane permeability, osmotic tolerance, cryoprotectant (CPA) toxicity, and mathematical modeling. This review provides a comprehensive evaluation of vitrification in bovine oocytes and IVP embryos by integrating biological, biophysical, and practical perspectives. It analyzes the cellular factors that limit cryotolerance and the advances in rational protocol design based on the quantitative analysis of membrane permeability and osmotic tolerance. The use of mathematical modeling, including temperature-dependent permeability data, has enabled the optimization of equilibration steps and reduced variability among laboratories. Moreover, strategies to mitigate cryodamage are discussed as promising but still inconsistent approaches. Technical improvements, including blastocoel collapse before vitrification and simplified warming systems for direct transfer, have also been evaluated for their potential to facilitate field application. Although vitrification remains biologically superior to slow freezing in terms of cryosurvival, its large‑scale implementation is limited by handling complexity and the lack of standardized direct‑transfer systems. Future progress in bovine cryopreservation will depend on combining enhanced cellular competence with biophysical optimization and standardized, reproducible procedures that can ensure not only improved post‑warming survival but also consistent pregnancy and calving outcomes in real production environments.

## Introduction

Cryopreservation of bovine oocytes and embryos is a key technology for modern animal reproduction and the preservation of genetic resources. The capacity to store and transport viable embryos has transformed breeding strategies, enabling global exchange of superior genetics, synchronization between donors and recipients, and reduced risk of disease transmission. The widespread adoption of *in vitro* embryo production (IVP) systems, together with genomic selection and oocyte pick‑up (OPU) technologies, has considerably increased the demand for efficient and reliable cryopreservation methods.

According to the most recent report of the International Embryo Technology Society (IETS), more than two million IVP bovine embryos were produced in 2024, exceeding the number of *in vivo*-derived embryos by almost sevenfold (Viana, 2025). This trend confirms the growing dominance of IVP systems in cattle breeding and their strategic importance for both dairy and beef industries. However, cryotolerance remains a major bottleneck in the IVP workflow. Only around 40% of IVP embryos are routinely cryopreserved and transferred, and pregnancy outcomes after thawing/warming remain inconsistent, particularly when compared with *in vivo*-derived embryos (Ealy et al., 2019; Hansen, 2020). These limitations are even more pronounced in oocytes, which remain the most difficult developmental stage to cryopreserve efficiently.

Vitrification is currently the preferred cryopreservation method for bovine oocytes and IVP embryos because it prevents intracellular ice crystal formation and limits chilling injury, the main sources of damage associated with conventional slow cooling. Success, however, depends on a precise balance among cooling and warming rates, cryoprotectant (CPA) concentration, and the osmotic tolerance of the cells (Saragusty and Arav, 2011). Bovine oocytes are particularly sensitive to cryodamage compared with embryos due to their large size and relatively low membrane permeability which limits water and CPA exchange during vitrification. In addition, their high lipid content, and highly organized intracellular architecture increase their susceptibility to osmotic, thermal, and oxidative injury, compromising normal fertilization and embryo development (reviewed in Dujíčková et al., 2020; Mogas et al., 2024). IVP embryos, although generally more cryotolerant than oocytes, still differ from *in vivo*-derived embryos in lipid metabolism, mitochondrial function, membrane composition, and developmental competence (Rizos et al., 2008). Thus, cryopreservation outcome depends not only on the vitrification protocol but also on the biological status of the cell prior to cooling. These intrinsic differences help explain why survival and pregnancy outcomes after transfer of cryopreserved IVP embryos remain variable and often less predictable than those obtained with *in vivo*-derived embryos.

Despite important progress, a universal and robust vitrification protocol for bovine oocytes and embryos is still lacking. Past improvements mainly resulted from empirical optimization of CPA composition, exposure time, temperature, and vitrification devices (reviewed in Ferré et al., 2020; Mogas et al., 2024). These adjustments have improved survival, but they have not eliminated the high sensitivity of bovine cells to subtle handling variations and inter‑laboratory reproducibility remains limited (reviewed in Mogas et al., 2024). Under field conditions, this limitation becomes critical: whereas direct‑transfer procedures are routinely applied for slow‑frozen embryos, comparable standardized systems for vitrified IVP embryos are still emerging. Reducing this gap is essential for wider and more consistent application of cryopreservation in breeding and germplasm conservation.

In this context, cryobiology and membrane transport analysis provide an essential basis for protocol design. The movement of water and CPAs across the plasma membrane, the osmotic tolerance of the cell, and the balance between adequate dehydration and CPA toxicity are key determinants of success. Integrating these biophysical concepts with biological determinants of oocyte and embryo competence offers a path toward more effective and reproducible vitrification strategies.

The aim of this review is to critically examine the current state of vitrification in bovine oocytes and IVP embryos by integrating biological and biophysical principles. The review discusses the main factors that define cryotolerance and explores new strategies to improve survival and consistency, from cellular preparation to methodological design and practical application.

## Biological constraints to cryopreservation of oocytes and embryos

The lower efficiency observed after oocyte vitrification compared with embryos is not caused by a single limitation but by several interacting factors affecting structure and function. Bovine oocytes are large cells with a low surface‑to‑volume ratio, which restricts the movement of water and CPAs across the plasma membrane. As a result, osmotic equilibration during vitrification and warming is slower and more difficult to control than in embryos, increasing the risk of excessive shrinkage, insufficient dehydration, and intracellular damage (reviewed in Dujíčková et al., 2020; Mogas et al., 2024).

Oocytes are also very fragile because their developmental competence depends on an organized intracellular architecture. The meiotic spindle is particularly sensitive to low temperature, and its depolymerization and chromosome scattering are frequent abnormalities after vitrification, even in oocytes that look morphologically normal (Garcia-Martinez et al., 2020; Morató et al., 2008a). Such changes are associated with abnormal fertilization and low embryo development. Other cytoskeletal elements, such as actin filaments and cortical granules, can also be disrupted, altering zona pellucida properties, sperm interaction, and pronuclear formation (Albarracin et al., 2005; Morató et al., 2008a). Ultrastructural studies of vitrified MII oocytes have described vacuolization, mitochondrial clustering, and reduced cortical granule content, confirming that cryodamage affects both structure and functional competence (Morató et al., 2008b).

A major factor for cryosensitivity is the high lipid content of bovine oocytes. Membrane lipids are highly responsive to temperature changes, and lipid phase transitions during cooling modify membrane fluidity, permeability, and integrity (Arav et al., 2000). The composition of these lipids, particularly cholesterol and the ratio of saturated to unsaturated fatty acids, explains inter‑ and intra‑species differences in cryotolerance and contributes to the particular fragility of bovine oocytes during vitrification (Jin et al., 2011; Seidel, 2006). In addition, the amount and distribution of cytoplasmic lipid droplets have a central role. In vitro maturation (IVM) usually increases lipid accumulation compared with *in vivo* maturation, leading to mitochondrial alterations, reduced metabolic efficiency, and a higher risk of chilling injury (Andrade Melo-Sterza and Poehland, 2021). Differences have also been described between bovine subspecies, as Bos taurus oocytes contain more lipid droplets than Bos indicus oocytes, which is relevant when considering breed-related variation in cryotolerance (Sudano et al., 2012). Mitochondrial function is closely linked to this lipid-dependent vulnerability. Vitrified oocytes often show altered mitochondrial potential, abnormal distribution, and increased formation of reactive oxygen species (ROS), indicating metabolic stress that can compromise ATP production and reduce developmental potential (Garcia-Martinez et al., 2020).

Several treatments have been tested to reduce lipid levels before vitrification, such as serum reduction or the use of delipidating agents (Chankitisakul et al., 2013; Spricigo et al., 2017). Their effects, however, are inconsistent and show that lipid content alone cannot explain post‑warming survival. Although lowering lipid levels may improve cryotolerance in some cases, it does not always preserve the cytoplasmic and nuclear coordination necessary for normal embryo development. In line with this, recent evidence using *in vivo* maturation through intrafollicular immature oocyte transfer (IFIOT) showed that, although vitrified oocytes reduced lipid accumulation and improved meiotic progression, chromatin integrity, and oxidative status, these improvements were still insufficient to restore embryo development after vitrification (Vieira Chaves et al., 2025).

**Figure 1:**
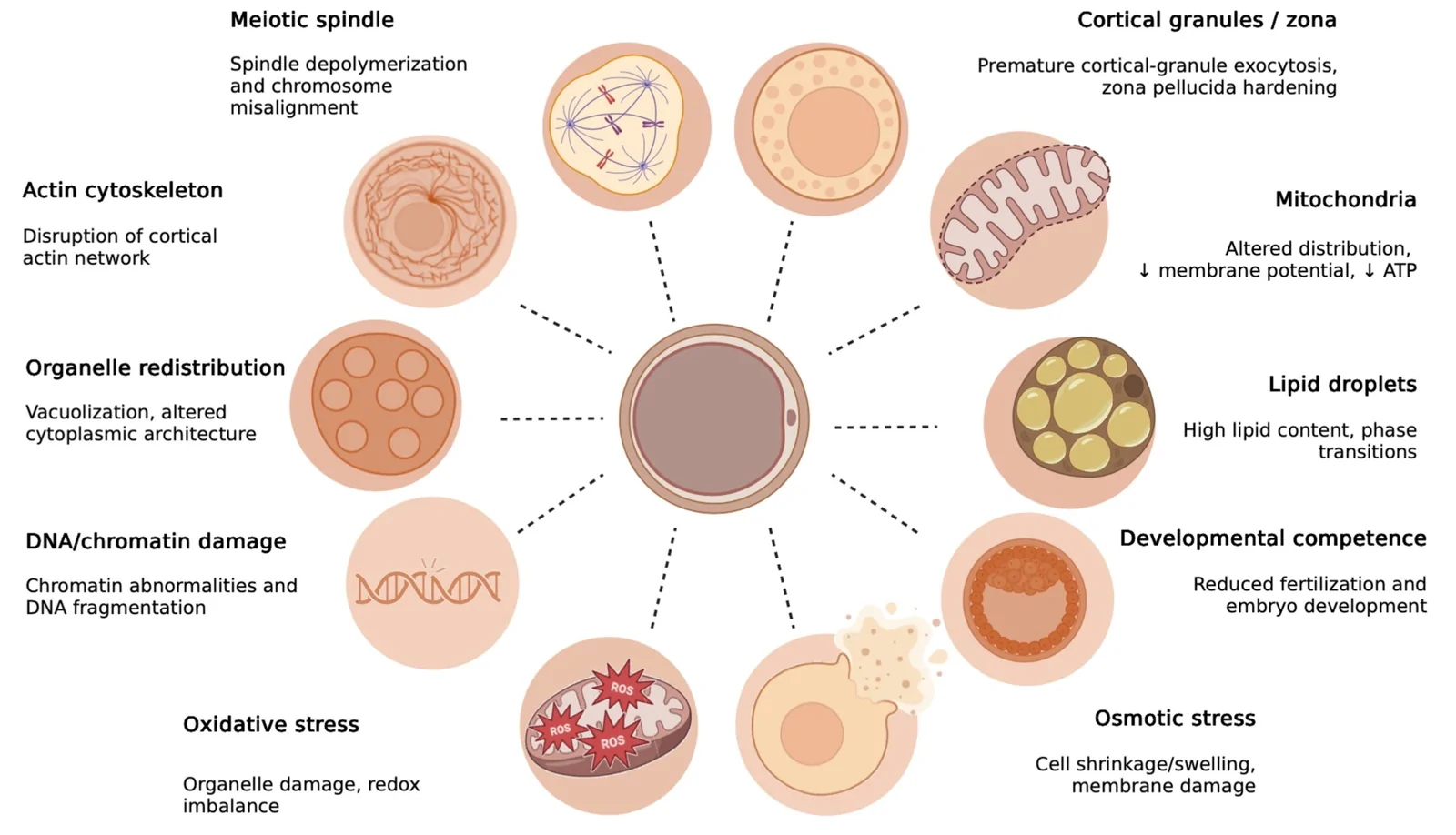
Cellular alterations associated with vitrification in oocytes. Vitrification induces multiple interconnected forms of cryoinjury, driven mainly by osmotic and oxidative stress, leading to membrane damage, organelle dysfunction, and altered cellular architecture. Key alterations include disruption of the meiotic spindle and cytoskeleton, mitochondrial dysfunction, lipid reorganization, and cortical granule exocytosis, all of which compromise cellular function and developmental competence. Embryos are generally more resistant than oocytes, but their cryotolerance depends closely on their biological quality. IVP embryos show lower survival than *in vivo*-derived embryos due to differences in metabolism, ultrastructure, and membrane composition (Rizos et al., 2008). Adjusting *in vitro* culture conditions, such as the use of serum-free systems and low oxygen tension, can improve lipid profiles and post-warming viability (Báez et al., 2021; Gomez et al., 2008). In addition, the use of metabolic modulators such as forskolin before vitrification has been associated with improved post-transfer pregnancy rates and enhanced cryotolerance of IVP embryos (Sanches et al., 2013).

The meiotic stage at which oocytes are vitrified represents an additional source of biological variability. Oocytes at the germinal vesicle (GV), germinal vesicle breakdown (GVBD), and metaphase II (MII) stages differ in both structural organization and developmental competence, and no single stage has been consistently identified as optimal for vitrification (Mo et al., 2014). MII oocytes are more competent but very sensitive due to the presence of the spindle (Chaves et al., 2017; Otoi et al., 1995), whereas GV oocytes avoid spindle damage but require maturation after warming (Zhou et al., 2010). Intermediate GVBD stages may represent a compromise, though results remain inconsistent (Spricigo et al., 2014). The cumulus cells also influence cryotolerance. While they support metabolism and signal exchange, they can slow water and CPA diffusion. Partial cumulus removal or addition of fresh cumulus cells after warming may balance protection and permeability (Ortiz-Escribano et al., 2016). Altogether, these observations illustrate that cryoinjury in bovine oocytes is a multifactorial process affecting both structural and functional components of the cell, as summarized in Figure 1.

Although morphological evaluation is still the main tool for embryo selection, it has clear limitations because static grading does not always reflect developmental competence. Studies using time‑lapse systems have demonstrated that embryo quality is a dynamic characteristic. Parameters such as an earlier first cleavage, a distinct lag‑phase, and the absence of irregular divisions are linked to higher blastocyst formation (Yaacobi-Artzi et al., 2022; Angel-Velez et al., 2023;). Transcriptomic analyses further confirm that different morphokinetic patterns correspond to specific gene‑expression profiles in the resulting blastocysts (Salilew-Wondim et al., 2021). Together, these data indicate that embryo quality is a multifactorial and time‑dependent process, and that morphology alone cannot accurately predict the true biological competence of IVP embryos.

The developmental stage at vitrification is another key determinant of success. Most studies and current field practice support vitrifying expanded blastocysts on Day 7 (D7), which usually show higher post‑warming survival and better pregnancy results than embryos vitrified on Day 8 (D8) or at earlier stages. Expanded and hatching blastocysts tend to tolerate vitrification better than non‑expanded embryos (Morató et al., 2010). However, this advantage is related not only to morphology but also to intrinsic embryo quality. Recent work has shown that embryos reaching the blastocyst stage later in culture (D8–D9) form a more heterogeneous and frequently compromised population, with reduced inner‑cell‑mass allocation, altered gene expression, and higher apoptosis (Kim et al., 2025). Thus, the better performance of D7 expanded blastocysts likely reflects both a more favorable stage for vitrification and a higher developmental competence of the embryos reaching this stage earlier.

In summary, these observations indicate that cryotolerance is highly dependent on the biological state of the oocyte or embryo prior to vitrification. Structural organization, metabolic activity, developmental stage and culture conditions determine how cells respond to osmotic stress and CPA exposure. Therefore, improving biological quality is not only beneficial but essential, as it directly affects the effectiveness of any cryopreservation protocol. This also implies that protocol optimization cannot be addressed independently of cell physiology, and that a more rational approach must integrate both biological and biophysical parameters (Jin et al., 2011; Edashige, 2016; Gomez et al., 2020).

## From empirical protocols to biophysical design

Despite the widespread use of vitrification in bovine oocytes and embryos, many protocols have traditionally been established by trial and error, with limited consideration of the underlying biophysical principles. As a result, key parameters such as CPA concentration, exposure time, and temperature are often selected without fully considering how cells respond to osmotic and chemical stress. This contributes to variability between laboratories and limits reproducibility.

The response of oocytes and embryos to vitrification is largely determined by the movement of water and CPAs across the plasma membrane. These processes depend on membrane permeability properties, including hydraulic conductivity (*L*_p_) and solute permeability (*P*_s_), which define the rate of water and CPA transport, respectively. Importantly, these parameters are not constant but vary with temperature, developmental stage, and species (Edashige, 2016). In bovine oocytes, this issue is especially critical because membrane permeability to water and CPAs is relatively low, and equilibration is therefore slower and more sensitive to temperature (Jin et al., 2011).

Fundamental studies have shown that, in oocytes and early-stage embryos, water and CPAs move predominantly by simple diffusion across the lipid bilayer, resulting in low permeability and high temperature dependence. In contrast, at later embryonic stages such as morula and blastocyst, permeability increases due to the presence of membrane channels, including aquaporins, which facilitate transport and reduce temperature sensitivity. In bovine oocytes specifically, both water and CPAs show relatively low permeability and high activation energy, indicating that diffusion remains the main transport mechanism. These properties explain why longer exposure times are required to achieve intracellular equilibration, but also why cells become more susceptible to osmotic stress and CPA toxicity (Jin et al., 2011).

Mathematical models, such as the two-parameter (2P) formalism (Kleinhans, 1998), allow the prediction of volumetric changes and intracellular CPA concentrations during exposure to CPA solutions. These models describe the dynamic balance between water efflux and CPA influx and can be used to simulate osmotic behavior under different conditions of temperature and exposure time. By integrating experimental permeability data, *in silico* predictions can identify conditions that minimize excessive cell shrinkage or swelling, which are major contributors to osmotic damage.

Using this approach, it has been demonstrated that equilibration steps can be optimized by adjusting exposure time according to the temperature-dependent permeability of the oocyte. For example, bovine MII oocytes exposed to equilibration solution (7.5% Ethylene Glycol + 7.5% DMSO) at 38.5 °C reach osmotic equilibrium significantly faster than at 25 °C, allowing shorter exposure times that reduce CPA toxicity while maintaining adequate intracellular CPA levels. These optimized conditions have been associated with improved spindle integrity, reduced DNA fragmentation, and higher blastocyst rates compared to conventional protocols (Garcia-Martinez et al., 2022).

A similar modeling-based strategy has been successfully applied to IVP bovine embryos. Martínez-Rodero et al. (2024a) used membrane permeability data and *in silico* predictions to define shorter equilibration times for D7 and D8 blastocysts at 25 °C and 38.5 °C. Their results showed that the optimized protocols at 38.5 °C improved post-warming performance, with re-expansion and hatching rates in D7 blastocysts comparable to fresh controls, and no differences between D7 and D8 blastocysts when temperature-specific protocols were used. Total cell number was also maintained in embryos vitrified at 38.5 °C but decreased after vitrification at 25 °C. These data support the idea that embryo vitrification can also benefit from protocol design based on permeability and osmotic response, rather than on empirical timing alone (Martinez-Rodero et al., 2024a).

Importantly, the value of this biophysical approach is not restricted to bovine species. In equine oocytes, where cryopreservation is also particularly challenging, permeability-based optimization has likewise shown that CPA equilibration is markedly influenced by temperature and occurs more slowly than often assumed (Gago et al., 2026a). When vitrification protocols are designed based on experimentally determined permeability parameters and optimized exposure conditions, post-warming oocytes show levels of ROS, glutathione content (GSH), and mitochondrial distribution and activity comparable to fresh controls (Gago et al., 2026b). This broader applicability reinforces the idea that model-based optimization can improve protocol design across species by reducing osmotic and toxic stress during CPA equilibration.

More advanced approaches consider cryopreservation as a balance between multiple forms of damage that occur during CPA loading and unloading. These include osmotic injury caused by excessive cell shrinkage or swelling, cumulative toxicity of CPA exposure and, in sensitive cells such as bovine oocytes, the possibility of chilling injury (Benson et al., 2012). The challenge, therefore, is not to minimize a single source of damage, but to find conditions that achieve sufficient intracellular CPA equilibration while keeping total injury as low as possible (Olver, 2025; Olver et al., 2023). In practice, this means balancing slower or stepwise procedures that reduce osmotic stress against the longer exposure times that may increase toxicity or chilling-related damage.

In this context, microfluidic systems offer a useful experimental platform because they allow more gradual and tightly controlled changes in extracellular osmolality than conventional manual handling. This is relevant because, unlike the sudden stepwise changes imposed by pipetting-based procedures, microfluidic devices can generate progressive concentration profiles around the cell, which may reduce osmotic shock and better preserve cell function during CPA equilibration (Zhao et al., 2017). In addition, microfluidic platforms can be combined with real-time image analysis to monitor cell volume during CPA exposure, creating the basis for feedback-controlled and individually optimized equilibration strategies for oocytes and embryos (Tu et al., 2022). However, despite their experimental potential, the implementation of these systems in routine bovine IVP laboratories remains challenging because they still require further simplification, standardization, and adaptation for large-scale application.

## Limiting cellular stress and damage

Cryopreservation induces a complex cascade of cellular stress responses that affect multiple structures and pathways simultaneously. Among these, oxidative stress is one of the main mechanisms underlying cryoinjury in both oocytes and embryos. The combined effects of low temperature, osmotic changes, and exposure to CPAs disrupt the balance between ROS production and the intrinsic antioxidant capacity of the cell, leading to alterations in redox homeostasis (Cao et al., 2022).

Mitochondria play a central role in cryodamage, as they are both a major source and a primary target of ROS. During vitrification and warming, mitochondrial dysfunction can increase ROS production, reduce ATP availability, and alter mitochondrial membrane potential, thereby compromising cellular activity and developmental competence. When ROS accumulate beyond physiological levels, they can also induce calcium release from the endoplasmic reticulum and damage proteins, lipids, DNA, and cytoskeletal structures (reviewed in (Gualtieri et al., 2021)). The meiotic spindle is highly sensitive to oxidative and thermal stress, and its depolymerization or abnormal organization is frequently observed after vitrification (Garcia-Martinez et al., 2020). Alterations in actin filaments and cortical structures have also been described, further affecting cellular integrity and fertilization capacity. Accordingly, excessive ROS accumulation has been consistently associated with reduced oocyte quality and impaired embryo development after cryopreservation (Garcia-Martinez et al., 2020). Therefore, it is necessary to reduce oxidative stress to preserve oocyte competence.

Given the central role of oxidative stress in cryodamage, several strategies have been explored to reduce its impact. The most common approach has been the supplementation with antioxidants during IVM, vitrification/warming, or post-warming culture. A wide range of compounds, including coenzyme Q10, vitamin E, L-carnitine, resveratrol, melatonin, and GSH, have been reported to reduce ROS levels, improve mitochondrial function, and partially restore developmental competence (reviewed in Mogas et al. (2024). In bovine oocytes, supplementation with GSH-Ethyl Ester during IVM improved mitochondrial distribution, reduced both cytoplasmic and mitochondrial ROS levels, and enhanced embryo development after vitrification and warming (Garcia-Martinez et al., 2020). Likewise, a short post-warming recovery with GSH improved embryo development, decreased the proportion of apoptotic cells, and reversed the detrimental effects of vitrification on the actin cytoskeleton (Olexikova et al., 2022).

Because cryoinjury affects several interconnected processes at the same time, including oxidative balance, cytoskeletal organization, DNA integrity, and metabolic activity, approaches acting on a single pathway often provide only partial protection. In this context, extracellular vesicles (EVs) have emerged as a particularly attractive strategy because they can transfer proteins, lipids, mRNAs, and miRNAs able to modulate several cellular pathways simultaneously. Their relevance in reproductive biology is increasingly recognized, especially in follicular communication and in the regulation of oocyte competence and embryo development (Gervasi et al., 2020).

Recent evidence in cattle indicates that EV supplementation during IVM can improve the response of oocytes to vitrification. In bovine oocytes, follicular fluid-derived EVs are internalized by cumulus-oocyte complexes and, when added during maturation, their effects on vitrification outcomes depend on follicular origin (Diaz-Muñoz et al., 2025b). EVs derived from large follicles were the most beneficial under vitrification conditions: they preserved normal spindle configuration, reduced DNA fragmentation to levels comparable with fresh controls, and supported blastocyst development after warming. In addition, embryos derived from vitrified oocytes supplemented with large-follicle EVs showed improved expansion and hatching rates, and inner cell mass cell numbers closer to those of non-vitrified controls (Diaz-Muñoz et al., 2025b). By contrast, EVs from small follicles did not provide the same protection, even though under non-stress conditions small-follicle EVs have often been associated with beneficial effects on cumulus expansion and embryo development (Hung et al., 2015; Silveira et al., 2017).

The protective action of EVs is likely related to their molecular cargo. This is supported by recent work using EVs derived from granulosa cells exposed to sublethal stress. In vitrified bovine oocytes, supplementation with EVs from oxidatively stressed granulosa cells improved subsequent embryo development and was associated with lower ROS levels and higher GSH content in the resulting blastocysts, indicating better redox regulation after warming (Diaz-Muñoz et al., 2025a). These EVs carried a distinct miRNA cargo, with 10 miRNAs more abundant in OS-EVs and predicted targets related to MAPK, PI3K-Akt, RAS, and Hippo signaling, as well as apoptosis, cell cycle regulation, migration, and cell growth, supporting the idea that their effects may extend beyond direct ROS scavenging (Diaz-Muñoz et al., 2026a). A related study further supports the idea that EV function highly depends on the physiological context of the donor cells. When bovine oocytes were matured in the presence of EVs from oxidatively stressed or heat-stressed granulosa cells, the two EV populations produced different outcomes (Diaz-Muñoz et al., 2026b). EVs from oxidatively stressed granulosa cells increased blastocyst yield, whereas EVs from heat-stressed granulosa cells were associated with reduced lipid content in the resulting blastocysts, with no changes in mitochondrial activity (Diaz-Muñoz et al., 2026b). These findings suggest that EVs do not act as a uniform supplement, but rather as context-dependent carriers whose cargo may target different aspects of embryo competence and cryotolerance, including redox homeostasis or lipid metabolism.

Evidence from other species, although still limited, points in the same general direction. In the domestic cat, follicular fluid EVs added before vitrification and/or during warming did not improve immediate survival, but enhanced meiotic resumption of vitrified oocytes, and only EV-treated vitrified oocytes reached MII. Proteomic analysis of these EVs identified proteins related to oxidative phosphorylation, oocyte meiosis, cytoskeletal regulation, calcium signaling, and stress responses, including chaperones associated with osmotic and thermal stress (Ferraz et al., 2020). Although this work was performed in a different species and at the immature oocyte stage, it supports the idea that follicular EVs may help preserve key functional properties of the oocyte after cryopreservation.

**Figure 2:**
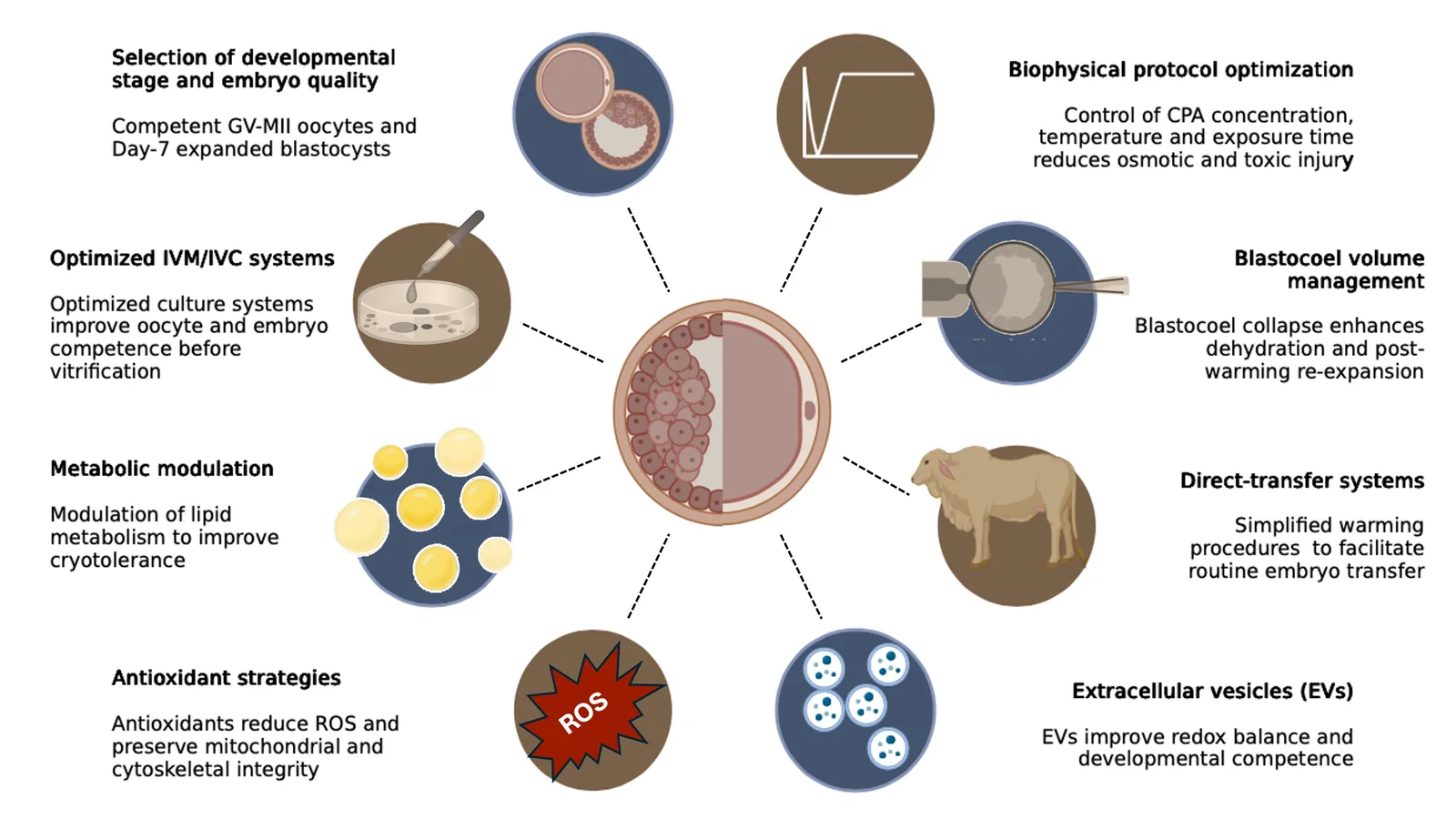
Main biological, technical, and biophysical strategies currently explored to improvevitrification efficiency and cryotolerance in bovine oocytes and in vitro-produced embryos. These approaches include a combination ofstrategies that improve cellular quality (culture conditions, modulation of lipid metabolism and oxidative stress, extracellular vesicle supplementation),optimize vitrification protocols (CPA composition, exposure conditions, blastocoel management), and facilitate field application (selection of competent stages and direct-transfer systems).

Overall, these findings suggest that EVs represent a more integrative strategy than conventional antioxidant supplementation alone. Rather than acting exclusively as ROS scavengers, EVs may simultaneously influence redox balance, spindle stability, DNA integrity, metabolic regulation, and developmental competence. However, current evidence also makes clear that their effects are not universal and depend on EV origin, follicular context, and donor-cell physiological state. The main biological, technical, and biophysical strategies currently explored to improve vitrification efficiency and cryotolerance in bovine oocytes and embryos are summarized in Figure 2.

## Technical and practical challenges on IVP embryo cryopreservation

Despite major progress in bovine embryo cryopreservation, the practical implementation of embryo cryopreservation in cattle is still limited by technical and translational constraints. The main challenge is no longer limited to post-warming survival, but rather to developing protocols that are simple, repeatable, and compatible with routine commercial use under commercial conditions (Ferré et al., 2020; Mogas et al., 2024; Sanches et al., 2017).

Vitrification generally provides better post-warming survival than slow freezing because it avoids ice crystal formation, which is particularly relevant for IVP embryos. However, its application under field conditions remains limited because it requires precise timing, handling in minimal volumes, and stepwise warming and CPA dilution, usually under stereomicroscope observation. These technical requirements make vitrification difficult to implement on a large scale, especially when many embryos must be processed or when transfer is performed outside the laboratory. For this reason, although vitrification is often the preferred cryopreservation method for IVP bovine embryos from a biological perspective, its large-scale use remains limited by logistics and by the lack of a simple and reliable direct transfer system (Dochi, 2019; Ferré et al., 2020; Mogas et al., 2024; Sanches et al., 2017).

### Artificial collapse of the blastocoel before cryopreservation

Among the technical strategies proposed to improve embryo cryopreservation, management of blastocoelic volume has become one of the most biologically and cryobiologically relevant. Expanded blastocysts contain a fluid-filled blastocoel that represents an additional water compartment during cryopreservation. This feature is relevant because insufficient reduction of blastocoelic volume before cooling has been associated with poorer post-warming outcomes. Previous studies have shown that blastocoel collapse or fluid removal before cryopreservation improves re-expansion and hatching, and may also influence cell proliferation, apoptosis, and DNA integrity, indicating that blastocoel volume is not only a morphological characteristic but also a functional determinant of cryotolerance (Chen et al., 2005; Marques et al., 2021; Martinez-Rodero et al., 2024b; Min et al., 2014).

By reducing the amount of fluid present in the blastocoel before exposure to CPAs and cooling, the embryo becomes less susceptible to ice-related damage during cryopreservation. Different approaches have been used to induce blastocoel collapse, including microneedle puncture (Chen et al., 2005), forced-assisted collapse (Min et al., 2014) together with direct aspiration of blastocoelic fluid (Martinez-Rodero et al., 2024b), and osmotic collapse (Jung et al., 2024). Although technically distinct, all of this approaches aim to facilitate dehydration before cryopreservation. In bovine embryos, this strategy has been associated with improved post-warming outcomes. Min et al. (2014) reported that forced blastocoel collapse before both slow freezing and vitrification of IVP bovine embryos increased survival and hatching, reduced apoptosis, and improved the expression of apoptosis-related genes, with better pregnancy-related outcomes after transfer of *in vivo*-derived embryos. Similarly, Marques et al. (2021) showed that blastocoel fluid removal, particularly when combined with melatonin supplementation during culture, improved post-warming re-expansion and hatching of vitrified IVP bovine blastocysts.

Beyond its effect on cryosurvival, artificial collapse may also have translational value in embryo selection programs. Martínez-Rodero et al. (2024b) showed that blastocoel fluid obtained after collapse of day 7 expanded IVP blastocysts provides cell-free DNA with sexing efficiency and accuracy comparable to trophectoderm biopsy, while yielding better post-warming re-expansion and hatching than biopsied vitrified embryos , with outcomes comparable to fresh controls. In addition, collapsed embryos showed a more favorable molecular profile, whereas biopsied embryos displayed increased BAX and altered ATP1B1 and AQP3 expression. This is particularly relevant for IVP bovine embryos, in which additional manipulations such as biopsy may further compromise cryotolerance. Therefore, blastocoel collapse should not be viewed only as a technical refinement to improve vitrification, but also as a minimally invasive strategy that may help integrate cryopreservation with embryo sexing/genotyping.

Similar concepts aimed at reducing blastocoelic fluid before cryopreservation have also been explored in slow freezing. For example, Jung et al. (2024) reported that osmotic pre-dehydration with 0.25 M sucrose before slow freezing improved post-thaw viability, reduced TUNEL-positive cells, and improved gene expression patterns in day-7 expanded bovine blastocysts. Although the mechanism differs from direct aspiration, both approaches support the idea that management of blastocoelic volume before cryopreservation can improve embryo survival by facilitating dehydration and reducing cryoinjury.

### Vitrification versus slow freezing: biological efficiency and field applicability

Vitrification and slow freezing represent two distinct strategies for bovine embryo cryopreservation, each with advantages and limitations in terms of biological performance and field applicability. Experimental evidence consistently shows that vitrification provides higher in vitro survival, re‑expansion, and hatching rates than slow freezing, both in intact and biopsied embryos (Arshad et al., 2021; Do and Taylor-Robinson, 2020; Najafzadeh et al., 2021; Gonzalez-Rodriguez et al., 2022). This is expected, as vitrification prevents the formation of ice crystals—one of the major causes of structural injury in conventional freezing. The meta‑analysis by Arshad et al. (2021) confirmed higher post‑warming survival and total cell number in vitrified embryos, but pregnancy rates after transfer remained similar between the two methods. This indicates that superior laboratory results do not necessarily ensure improved field outcomes.

From a practical point of view, the main strength of slow freezing is its compatibility with direct transfer (DT), which avoids post‑thaw dilution steps and allows simple embryo handling under farm conditions (Dochi, 2019). Vitrification, in contrast, requires very precise timing, small‑volume manipulation, and multiple warming steps, making it less convenient for large‑scale or field use (Ferré et al., 2020; Mogas et al., 2024). Consequently, although vitrification is biologically superior, slow freezing remains more accessible for routine programs.

Comparative studies illustrate this contrast between biological efficiency and practical applicability. In commercial conditions, pregnancy results from vitrified and direct‑transfer (DT) frozen IVP embryos were often similar, even though vitrification produced higher in vitro survival. Sanches et al. (2016) reported conception rates of 51.4 % for fresh embryos, 35.9 % for vitrified embryos, and 40.2 % for frozen embryos transferred directly. Although fresh embryos yielded the highest pregnancy rates, DT of slow‑frozen embryos provided similar results to vitrification, while allowing a simpler and faster procedure compatible with field work. Likewise, Gómez et al. (2020), working with a one‑step DT freezing system for IVP embryos produced under chemically defined conditions, observed that vitrification gave higher in vitro survival, but birth rates after transfer of Day 7 blastocysts did not differ significantly among fresh, frozen, and vitrified embryos. Altogether, these studies show that the lower cryobiological performance of slow freezing can be partly balanced by its operational simplicity and ease of use under farm conditions.

At the same time, the field success of slow freezing is highly dependent on embryo quality. Only high‑quality embryos, usually classified as IETS Grade 1, are considered suitable for direct transfer if acceptable pregnancy rates are to be obtained (Dochi, 2019; Valente et al., 2022). This requirement is especially relevant for IVP embryos, which are generally more cryosensitive than in vivo‑derived ones. Cryosurvival is also influenced by the production system. Ishii et al. (Ishii et al., 2024) reported that embryos produced in chemically defined, serum‑free media showed better post‑thaw viability and lower incidence of miscarriage after Day 40 than those cultured with serum. These findings confirm that the outcome of cryopreservation depends not only on the freezing method itself, but also on the intrinsic biological quality of the embryos before freezing. Several strategies have been tested to improve cryoresistance and DT efficiency in slow freezing, including supplementation with antioxidants and osmolytes such as carnosine, L‑proline, or ROCK inhibitor, which reduce oxidative stress and apoptosis (Carrascal-Triana et al., 2022; Ishii et al., 2024; Jung et al., 2025). Likewise, sucrose pre-equilibration has been used to reduce blastocoelic volume before freezing (Jung et al., 2024). Although these strategies remain supported mainly by *in vitro* data, they indicate that the biological efficiency of slow freezing can still be improved.

In parallel, considerable effort has been directed toward reducing the translational gap of vitrification by adapting it to direct transfer or in-straw warming systems. The objective is to retain the superior cryobiological performance of vitrification while simplifying warming and transfer under field conditions. Early studies demonstrated the technical feasibility of this approach (Inaba et al., 2011; Morató and Mogas, 2014), and more recent work with systems such as VitTrans has reported promising results. Martínez-Rodero et al. (2021) showed that supplementation of the VitTrans vitrification medium with exopolysaccharide ID1 improved post-warming embryo development and yielded survival rates comparable to Cryotop. Likewise, Gonzalez-Rodríguez et al. (2022) showed that vitrification combined with one-step warming or in-straw warming can be compatible with embryo transfer, at least in biopsied *in vivo*-derived embryos. Although these studies support the feasibility of simplified warming approaches, validation under large-scale commercial conditions remains limited. Therefore, vitrification‑based DT has not yet reached the robustness, consistency or adoption level achieved by conventional DT after slow freezing.

A major unresolved issue for both systems is standardization. Reported pregnancy rates vary markedly among studies and commercial settings, reflecting not only differences in cryopreservation procedures, but also variation in embryo production, embryo selection, recipient management, and transfer conditions. Recipient-related factors are especially important. Hansen (Hansen, 2020) emphasized that embryo transfer outcomes are still markedly conditioned by recipient factors and uterine environment, even when early developmental failures are bypassed. Similarly, the recent Korean field study showed that pregnancy rates after transfer did not differ significantly according to embryo type or recipient parity, but did differ among farms, and these differences were associated with recipient metabolic profile and feed management rather than with embryo category alone (Jung et al., 2026). These data highlight that part of the variability often attributed to the cryopreservation method may reflect differences in recipient quality and farm management.

In summary, vitrification remains the biologically superior approach for bovine IVP embryos, whereas slow freezing is more practical for routine field use, although its final performance depends much more heavily on embryo quality and remains highly influenced by recipient and farm variability. Therefore, the central challenge is not only to improve embryo survival after warming, but to translate that improvement into consistent pregnancy and calving outcomes under commercial conditions. In this context, future progress will depend on combining the biological advantages of vitrification with the practical simplicity of direct transfer, while integrating embryo quality, recipient condition, and farm management as essential components of the cryopreservation process.

## Conclusions and future perspectives

Vitrification has become the most biologically effective cryopreservation method for bovine oocytes and IVP embryos because it minimizes ice crystal formation and generally improves post‑warming survival compared with slow freezing. However, its efficiency remains highly dependent on the intrinsic quality of the oocyte or embryo before cryopreservation. This limitation is particularly evident in bovine systems, where high lipid content, cytoplasmic fragility, mitochondrial dysfunction, and changes related to IVP conditions still restrict cryotolerance. The outcome of cryopreservation, therefore, cannot be determined by protocol design alone.

A major advance in the field has been the shift from empirical optimization toward a rational framework based on membrane permeability, osmotic behavior, cryoprotectant toxicity, and mathematical modeling. This approach provides a more solid basis to reduce variability and to adapt vitrification procedures to the specific biological properties of each cell type and developmental stage. At the same time, strategies aimed at improving cellular competence before vitrification—such as optimized maturation systems, antioxidant treatments, or the use of extracellular vesicles—represent promising complementary tools, although their effects are still partial and often inconsistent among laboratories.

Beyond laboratory optimization, the next step is to define cryopreservation success in a broader and more practical context. For bovine embryos, post‑warming survival alone is not sufficient if it does not result in consistent pregnancy and calving rates. Consequently, future progress must integrate cryobiological efficiency with embryo quality, recipient factors, and the operational requirements of commercial embryo‑transfer programs.

Overall, advances in bovine cryopreservation will depend on a combined strategy: improving the intrinsic competence of oocytes and embryos, designing vitrification protocols on a biophysical rather than purely empirical basis, and validating simplified transfer systems under field conditions. Integrating cell biology, cryobiology, and applied reproduction will be essential to transform laboratory success into reliable reproductive outcomes in cattle production.

## Data Availability

Data sharing is not applicable to this article as no new data were created or analysed in this study.

## References

[B001] Albarracín JL, Morató R, Rojas C, Mogas T (2005). Effects of vitrification in open pulled straws on the cytology of in vitro matured prepubertal and adult bovine oocytes. Theriogenology.

[B002] Andrade Melo-Sterza F, Poehland R (2021). Lipid metabolism in bovine oocytes and early embryos under in vivo, in vitro, and stress conditions. Int J Mol Sci.

[B003] Angel-Velez D, De Coster T, Azari-Dolatabad N, Fernández-Montoro A, Benedetti C, Pavani K, Van Soom A, Bogado Pascottini O, Smits K (2023). Embryo morphokinetics derived from fresh and vitrified bovine oocytes predict blastocyst development and nuclear abnormalities. Sci Rep.

[B004] Arav A, Pearl M, Zeron Y (2000). Does membrane lipid profile explain chilling sensitivity and membrane lipid phase transition of spermatozoa and oocytes?. Cryo Letters.

[B005] Arshad U, Sagheer M, Gonzalez-Silvestry FB, Hassan M, Sosa F (2021). Vitrification improves in-vitro embryonic survival in Bos taurus embryos without increasing pregnancy rate post embryo transfer when compared to slow-freezing: A systematic meta-analysis. Cryobiology.

[B006] Báez F, de Brun V, Rodríguez-Osorio N, Viñoles C (2021). 32 Low oxygen tension during in vitro oocyte maturation and fertilisation improves cryotolerance of bovine blastocysts produced in vitro. Reprod Fertil Dev.

[B007] Benson JD, Kearsley AJ, Higgins AZ (2012). Mathematical optimization of procedures for cryoprotectant equilibration using a toxicity cost function. Cryobiology.

[B008] Cao B, Qin J, Pan B, Qazi IH, Ye J, Fang Y, Zhou G (2022). Oxidative Stress and Oocyte Cryopreservation: Recent Advances in Mitigation Strategies Involving Antioxidants. Cells.

[B009] Carrascal-Triana EL, Zolini AM, de King AR, Penitente-Filho JM, Hansen PJ, Torres CAA, Block J (2022). Effect of addition of ascorbate, dithiothreitol or a caspase-3 inhibitor to cryopreservation medium on post-thaw survival of bovine embryos produced in vitro. Reprod Domest Anim.

[B010] Chankitisakul V, Somfai T, Inaba Y, Techakumphu M, Nagai T (2013). Supplementation of maturation medium with L-carnitine improves cryo-tolerance of bovine in vitro matured oocytes. Theriogenology.

[B011] Chaves DF, Corbin E, Almiñana C, Locatelli Y, Souza-Fabjan JMG, Bhat MH, Freitas VJF, Mermillod P (2017). Vitrification of immature and in vitro matured bovine cumulus-oocyte complexes: effects on oocyte structure and embryo development. Livest Sci.

[B012] Chen S-U, Lee T-H, Lien Y-R, Tsai Y-Y, Chang L-J, Yang Y-S (2005). Microsuction of blastocoelic fluid before vitrification increased survival and pregnancy of mouse expanded blastocysts, but pretreatment with the cytoskeletal stabilizer did not increase blastocyst survival. Fertil Steril.

[B013] Diaz-Muñoz J, Cajas Yulia N, Cañón-Beltrán K, Arevalo L, Gago S, Lopez-Bejar M (2025). Extracellular vesicles from oxidatively stressed granulosa cells improve embryo development and redox balance after vitrification of in vitro matured bovine oocytes. Anim Reprod.

[B014] Diaz-Muñoz J, Cañón-Beltrán K, Cajas YN, Gago S, Iniesta-Cuerda M, Soler AJ, Rizos D, Mogas T (2025). Enhancing developmental potential of vitrified in vitro matured bovine oocytes using extracellular vesicles from large follicles. Sci Rep.

[B015] Diaz-Muñoz J, Cajas YN, Cañón-Beltrán K, Arévalo L, Gago S, Iniesta-Cuerda M (2026). Changes in the microRNA cargo of granulosa cell-derived extracellular vesicles under oxidative stress in a bovine model. Biol Res.

[B016] Diaz-Muñoz J, Gago S, Cajas YN, Cañon-Beltran K, Lopez-Bejar M, Rizos D, Mogas T (2026). Extracellular vesicles from heat and oxidative stressed granulosa cells modulate bovine in vitro embryo development and quality. Reprod Fertil Dev.

[B017] Do V, Taylor-Robinson A (2020). Cryopreservation of in vitro-produced bovine embryos by vitrification: in pursuit of a simplified, standardized procedure that improves pregnancy rates to promote cattle industry use. Biotechnol Anim Husb.

[B018] Dochi O (2019). Direct transfer of frozen-thawed bovine embryos and its application in cattle reproduction management. J Reprod Dev.

[B019] Dujíčková L, Makarevich AV, Olexikova L, Kubovicova E, Strejcek F (2020). Methodological approaches for vitrification of bovine oocytes. Zygote.

[B020] Ealy AD, Wooldridge LK, McCoski SR (2019). Post-transfer consequences of in vitro-produced embryos in cattle. J Anim Sci.

[B021] Edashige K (2016). The movement of water and cryoprotectants across the plasma membrane of mammalian oocytes and embryos and its relevance to vitrification. J Reprod Dev.

[B022] Ferraz AM, Fujihara M, Nagashima JB, Noonan MJ, Inoue-Murayama M, Songsasen N (2020). Follicular extracellular vesicles enhance meiotic resumption of domestic cat vitrified oocytes. Sci Rep.

[B023] Ferré LB, Kjelland ME, Taiyeb AM, Campos-Chillon F, Ross PJ (2020). Recent progress in bovine in vitro-derived embryo cryotolerance: impact of in vitro culture systems, advances in cryopreservation and future considerations. Reprod Domest Anim.

[B024] Gago S, García-Martínez T, Diaz-Muñoz J, Acacio M, Catalán J, Miró J, Higgins AZ, Costa-Borges N, Mogas T (2026). Water and cryoprotectant permeability of mature equine oocytes: experimental measurements and in silico predictions. Sci Rep.

[B025] Gago S, Vendrell-Flotats M, Diaz-Muñoz J, Soriano-Moya M, Acacio M, Mestres E (2026). Oxidative and mitochondrial status of vitrified equine in vitro matured oocytes using temperature-specific equilibration protocols. Reprod Domest Anim.

[B026] García-Martinez T, Vendrell-Flotats M, Martinez-Rodero I, Ordonez-Leon EA, Alvarez-Rodriguez M, Lopez-Bejar M, Yeste M, Mogas T (2020). Glutathione ethyl ester protects in vitro-maturing bovine oocytes against oxidative stress induced by subsequent vitrification/warming. Int J Mol Sci.

[B027] García-Martinez T, Martinez-Rodero I, Roncero-Carol J, Yanez-Ortiz I, Higgins AZ, Mogas T (2022). Impact of equilibration duration combined with temperature on the outcome of bovine oocyte vitrification. Theriogenology.

[B028] Gervasi MG, Soler AJ, Gonzalez-Fernandez L, Alves MG, Oliveira PF, Martin-Hidalgo D (2020). Extracellular Vesicles, the Road toward the Improvement of ART Outcomes. Animals.

[B029] Gómez E, Carrocera S, Martín D, Pérez-Jánez JJ, Prendes J, Prendes JM, Vázquez A, Murillo A, Gimeno I, Muñoz M (2020). Efficient one-step direct transfer to recipients of thawed bovine embryos cultured in vitro and frozen in chemically defined medium. Theriogenology.

[B030] Gomez E, Rodriguez A, Munoz M, Caamano JN, Hidalgo CO, Moran E, Facal N, Díez C (2008). Serum free embryo culture medium improves in vitro survival of bovine blastocysts to vitrification. Theriogenology.

[B031] González-Rodríguez N, Martinez-Rodero I, Scherzer J, Jung S, Reichenbach M, Zablotski Y, Otzdorff C, Zerbe H, Mogas T (2022). Vitrification and in-straw warming do not affect pregnancy rates of biopsied bovine embryos. Theriogenology.

[B032] Gualtieri R, Kalthur G, Barbato V, Di Nardo M, Adiga SK, Talevi R (2021). Mitochondrial dysfunction and oxidative stress caused by cryopreservation in reproductive cells. Antioxidants.

[B033] Hansen PJ (2020). The incompletely fulfilled promise of embryo transfer in cattle-why aren’t pregnancy rates greater and what can we do about it?. J Anim Sci.

[B034] Hung W-T, Hong X, Christenson LK, McGinnis LK (2015). Extracellular Vesicles from Bovine Follicular Fluid Support Cumulus Expansion. Biol Reprod.

[B035] Inaba Y, Aikawa Y, Hirai T, Hashiyada Y, Yamanouchi T, Misumi K, Ohtake M, Somfai T, Kobayashi S, Saito N, Matoba S, Konishi K, Imai K (2011). In-straw cryoprotectant dilution for bovine embryos vitrified using Cryotop. J Reprod Dev.

[B036] Ishii T, Mori-Kobayashi K, Nakamura S, Ohkura S, Matsuyama S (2024). Carnosine supplementation in cryopreservation solution improved frozen-thawed bovine embryo viability. J Reprod Dev.

[B037] Jin B, Kawai Y, Hara T, Takeda S, Seki S, Nakata Y, Matsukawa K, Koshimoto C, Kasai M, Edashige K (2011). Pathway for the movement of water and cryoprotectants in bovine oocytes and embryos. Biol Reprod.

[B038] Jung S, Sul H, Oh D, Jung Y-G, Lee J, Hyun S-H (2024). Slow freezing cryopreservation of Korean bovine blastocysts with an additional sucrose pre-equilibration step. Front Vet Sci.

[B039] Jung S, Jung Y, Sul H, Jung Y-G, Ham J, Oh D, Lee J, Hyun SH (2025). L-proline supplementation in the freezing medium enhances the viability and quality of bovine blastocysts after slow freezing and thawing. Theriogenology.

[B040] Jung S, Yang H, Jung Y, Lee M, Sul H, Jung Y-G, Lee J, Hyun SH (2026). Optimization of the embryo transfer technique in the korean native cattle: effects of key influencing factors. Animals.

[B041] Kim T-G, Choe Y-H, Kim S-H, Lee S-Y, Jang M, Yun S-H, Kim SJ, Lee SL, Lee WJ (2025). Increased apoptosis in late-developing *in vitro* fertilized bovine blastocysts decreases successful pregnancy. Anim Biosci.

[B042] Kleinhans FW (1998). Membrane permeability modeling: Kedem-Katchalsky vs a two-parameter formalism. Cryobiology.

[B043] Marques TC, Santos E, Diesel TO, Martins CF, Cumpa HCB, Leme LO, Dode MAN, Alves BG, Costa FPH, Oliveira EB, Gambarini ML (2021). Blastocoel fluid removal and melatonin supplementation in the culture medium improve the viability of vitrified bovine embryos. Theriogenology.

[B044] Martinez-Rodero I, Diaz-Munoz J, Higgins AZ, Lopez Bejar M, Mogas T, Garcia-Martinez T (2024). In silico-designed vitrification protocols: an approach to improve survival of in vitro produced-bovine embryos. Reproduction.

[B045] Martínez-Rodero I, Garcia-Martinez T, Ordonez-Leon EA, Vendrell-Flotats M, Olegario Hidalgo C, Esmoris J, Mendibil X, Azcarate S, López-Béjar M, Yeste M, Mogas T (2021). A shorter equilibration period improves post-warming outcomes after vitrification and in straw dilution of in vitro-produced bovine embryos. Biology.

[B046] Martínez-Rodero I, Salas-Huetos A, Diaz-Munoz J, Ordonez-Leon EA, Garcia-Martinez T, Yeste M, Olegario Hidalgo C, Mogas T (2024). Blastocoel fluid aspiration improves vitrification outcomes and produces similar sexing results of in vitro-produced cattle embryos compared to microblade biopsy. Theriogenology.

[B047] Min SH, Kim JW, Lee YH, Park SY, Jeong PS, Yeon JY, Park H, Chang KT, Koo DB (2014). Forced collapse of the blastocoel cavity improves developmental potential in cryopreserved bovine blastocysts by slow-rate freezing and vitrification. Reprod Domest Anim.

[B048] Mo XH, Fu XW, Yuan DS, Wu GQ, Jia BY, Cheng KR, Du M, Zhou YH, Yue MX, Hou YP, Li JJ, Zhu SE (2014). Effect of meiotic status, cumulus cells and cytoskeleton stabilizer on the developmental competence of ovine oocytes following vitrification. Small Rumin Res.

[B049] Mogas T, Garcia-Martinez T, Martinez-Rodero I (2024). Methodological approaches in vitrification: enhancing viability of bovine oocytes and in vitro-produced embryos. Reprod Domest Anim.

[B050] Morató R, Izquierdo D, Paramio MT, Mogas T (2008). Cryotops versus open-pulled straws (OPS) as carriers for the cryopreservation of bovine oocytes: effects on spindle and chromosome configuration and embryo development. Cryobiology.

[B051] Morató R, Mogas T, Maddox-Hyttel P (2008). Ultrastructure of bovine oocytes exposed to Taxol prior to ops vitrification. Mol Reprod Dev.

[B052] Morató R, Izquierdo D, Paramio MT, Mogas T (2010). Survival and apoptosis rates after vitrification in cryotop devices of in vitro-produced calf and cow blastocysts at different developmental stages. Reprod Fertil Dev.

[B053] Morató R, Mogas T (2014). New device for the vitrification and in-straw warming of in vitro produced bovine embryos. Cryobiology.

[B054] Najafzadeh V, Bojsen-Moller Secher J, Pihl M, Ærenlund A, Jørgensen N, Jensen KK, Jensen MT, Fenner MF, Strøbech L, Hyttel P (2021). Vitrification yields higher cryo-survival rate than slow freezing in biopsied bovine in vitro produced blastocysts. Theriogenology.

[B055] Olexiková L, Dujickova L, Makarevich AV, Bezdicek J, Sekaninova J, Nesvadbova A, Chrenek P (2022). Glutathione during Post-Thaw Recovery Culture Can Mitigate Deleterious Impact of Vitrification on Bovine Oocytes. Antioxidants.

[B056] Olver DJ, Heres P, Paredes E, Benson JD (2023). Rational synthesis of total damage during cryoprotectant equilibration: modelling and experimental validation of osmomechanical, temperature, and cytotoxic damage in sea urchin (*Paracentrotus lividus*) oocytes. PeerJ.

[B057] Olver DJ (2025). Mathematical cryobiology: modelling cellular physiology and population kinetics..

[B058] Ortiz-Escribano N, Smits K, Piepers S, Van den Abbeel E, Woelders H, Van Soom A (2016). Role of cumulus cells during vitrification and fertilization of mature bovine oocytes: effects on survival, fertilization, and blastocyst development. Theriogenology.

[B059] Otoi T, Yamamoto K, Koyama N, Suzuki T (1995). In vitro fertilization and development of immature and mature bovine oocytes cryopreserved by ethylene glycol with sucrose. Cryobiology.

[B060] Rizos D, Clemente M, Bermejo-Alvarez P, de La Fuente J, Lonergan P, Gutierrez-Adan A (2008). Consequences of in vitro culture conditions on embryo development and quality. Reprod Domest Anim.

[B061] Salilew-Wondim D, Tesfaye D, Rings F, Held-Hoelker E, Miskel D, Sirard M-A, Tholen E, Schellander K, Hoelker M (2021). The global gene expression outline of the bovine blastocyst: reflector of environmental conditions and predictor of developmental capacity. BMC Genomics.

[B062] Sanches BV, Lunardelli PA, Tannura JH, Cardoso BL, Pereira MH, Gaitkoski D, Basso AC, Arnold DR, Seneda MM (2016). A new direct transfer protocol for cryopreserved IVF embryos. Theriogenology.

[B063] Sanches BV, Marinho LS, Filho BD, Pontes JH, Basso AC, Meirinhos ML, Silva-Santos KC, Ferreira CR, Seneda MM (2013). Cryosurvival and pregnancy rates after exposure of IVF-derived Bos indicus embryos to forskolin before vitrification. Theriogenology.

[B064] Sanches BV, Zangirolamo AF, Silva NC, Morotti F, Seneda MM (2017). Cryopreservation of in vitro-produced embryos: challenges for commercial implementation. Anim Reprod.

[B065] Saragusty J, Arav A (2011). Current progress in oocyte and embryo cryopreservation by slow freezing and vitrification. Reproduction.

[B066] Seidel GE (2006). Modifying oocytes and embryos to improve their cryopreservation. Theriogenology.

[B067] Silveira JC, Andrade GM, del Collado M, Sampaio RV, Sangalli JR, Silva LA, Pinaffi FVL, Jardim IB, Cesar MC, Nogueira MFG, Cesar ASM, Coutinho LL, Pereira RW, Perecin F, Meirelles FV (2017). Supplementation with small-extracellular vesicles from ovarian follicular fluid during in vitro production modulates bovine embryo development. PLoS One.

[B068] Sprícigo JF, Morais K, Ferreira AR, Machado GM, Gomes AC, Rumpf R, Franco MM, Dode MA (2014). Vitrification of bovine oocytes at different meiotic stages using the Cryotop method: assessment of morphological, molecular and functional patterns. Cryobiology.

[B069] Sprícigo JF, Morató R, Arcarons N, Yeste M, Dode MA, Lopez-Bejar M, Mogas T (2017). Assessment of the effect of adding L-carnitine and/or resveratrol to maturation medium before vitrification on in vitro-matured calf oocytes. Theriogenology.

[B070] Sudano MJ, Santos VG, Tata A, Ferreira CR, Paschoal DM, Machado R, Buratini J, Eberlin MN, Landim-Alvarenga FD (2012). Phosphatidylcholine and sphingomyelin profiles vary in Bos taurus indicus and Bos taurus taurus in vitro- and in vivo-produced blastocysts. Biol Reprod.

[B071] Tu F, Bhat M, Benson JD (2022). Real-time computer assisted measurement of oocyte and embryo volume for assessment of transport parameters. Cryobiology.

[B072] Valente RS, Marsico TV, Sudano MJ (2022). Basic and applied features in the cryopreservation progress of bovine embryos. Anim Reprod Sci.

[B073] Viana JH (2025). 2023 Statistics of embryo production and transfer in domestic farm animals. Embryo Technol Newsl.

[B074] Vieira Chaves JE, Nicolás ACCV, Kussano NR, Pimenta LKL, Oliveira Silva VA, Souza E Castro AC, Gomes ACMM, de França E Melo L, Dode MAN, Sprícigo JFW (2025). Intrafollicular immature oocyte transfer (IFIOT) for IN VIVO maturation of vitrified bovine oocytes. Theriogenology.

[B075] Yaacobi-Artzi S, Kalo D, Roth Z (2022). Association between the morphokinetics of in-vitro-derived bovine embryos and the transcriptomic profile of the derived blastocysts. PLoS One.

[B076] Zhao G, Zhang Z, Zhang Y, Chen Z, Niu D, Cao Y, He X (2017). A microfluidic perfusion approach for on-chip characterization of the transport properties of human oocytes. Lab Chip.

[B077] Zhou XL, Al Naib A, Sun DW, Lonergan P (2010). Bovine oocyte vitrification using the Cryotop method: effect of cumulus cells and vitrification protocol on survival and subsequent development. Cryobiology.

